# OMRT-7. Angiogenesis inhibitors strongly synergize with therapeutics targeting tumor metabolism

**DOI:** 10.1093/noajnl/vdab070.032

**Published:** 2021-07-05

**Authors:** Sunada Khadka

**Affiliations:** UT MD Anderson Cancer Center, Houston, TX, USA. MD Anderson UT Health GSBS, Houston, TX, USA

## Abstract

Angiogenesis inhibition has become a mainstay of oncology despite having fallen short of its early promise. As originally envisioned, angiogenesis inhibition would cut off the blood supply, deprive tumor cells of key nutrients, leading to their death. In practice, while there is evidence that tumors under angiogenesis treatment do in fact exhibit some degree of metabolic stress, this is stress is not sufficient to induce significant cancer cell death. We posit that the full potential of angiogenesis inhibition can be realized by the combination of angiogenesis inhibition with emerging tumor metabolism targeting therapies. Because tumors under angiogenesis inhibition are already in a state of nutrient stress, the effects of metabolically targeted therapies such as amino acid depletion (e.g. asparginase, methionine restriction), inhibitors of stress adaption (AMPK and GCN2 inhibitors) or energy metabolism (e.g. IACS-010759, Metformin, POMHEX) stand to dramatically increase in potency whilst remaining selective for (angiogenic) tumor versus (non-angiogenic) normal tissue. Here, we provide proof-of-principal for this thesis. First, we performed metabolomic profiling of angiogenesis-inhibited tumors, which corroborates a state of nutrient stress in angiogenesis-inhibited tumors. Second, we demonstrate dramatic anti-neoplastic synergy (effectively curing of xenografted tumor-bearing mice, irrespective of initial tumor size), without enhanced adverse toxicities, between the OxPhos inhibitor IACS-010759 and the angiogenesis tyrosine kinase inhibitor, Tivozanib. The same results were recapitulated with the anti-VEGFA antibody, Avastin, and the OxPhos inhibitor could be substituted with the Enolase inhibitor HEX, with similar effects. The synergy was observed in a broad range of tumor types, even those without clear genetic susceptibilities. Together, these results suggest that angiogenesis inhibitors synergize broadly with cancer therapies targeting metabolism, allowing the realization of the full potential of these previously disappointing drugs. Our results warrant systematic combination clinical trials between angiogenesis inhibitors and established, as well as emerging anti-metabolic cancer therapies.

